# Supplementary material for: Longitudinal analysis at three oral sites links oral microbiota to clinical outcomes in allogeneic hematopoietic stem-cell transplant
Source: Microbiol Spectr. 2023 Nov 15;11(6):e02910-23. doi: 10.1128/spectrum.02910-23 (PMC10714774; doi:10.1128/spectrum.02910-23)
Supplement: File S1 — Timelines of antibiotic usage. [file spectrum.02910-23-s0001.pdf]

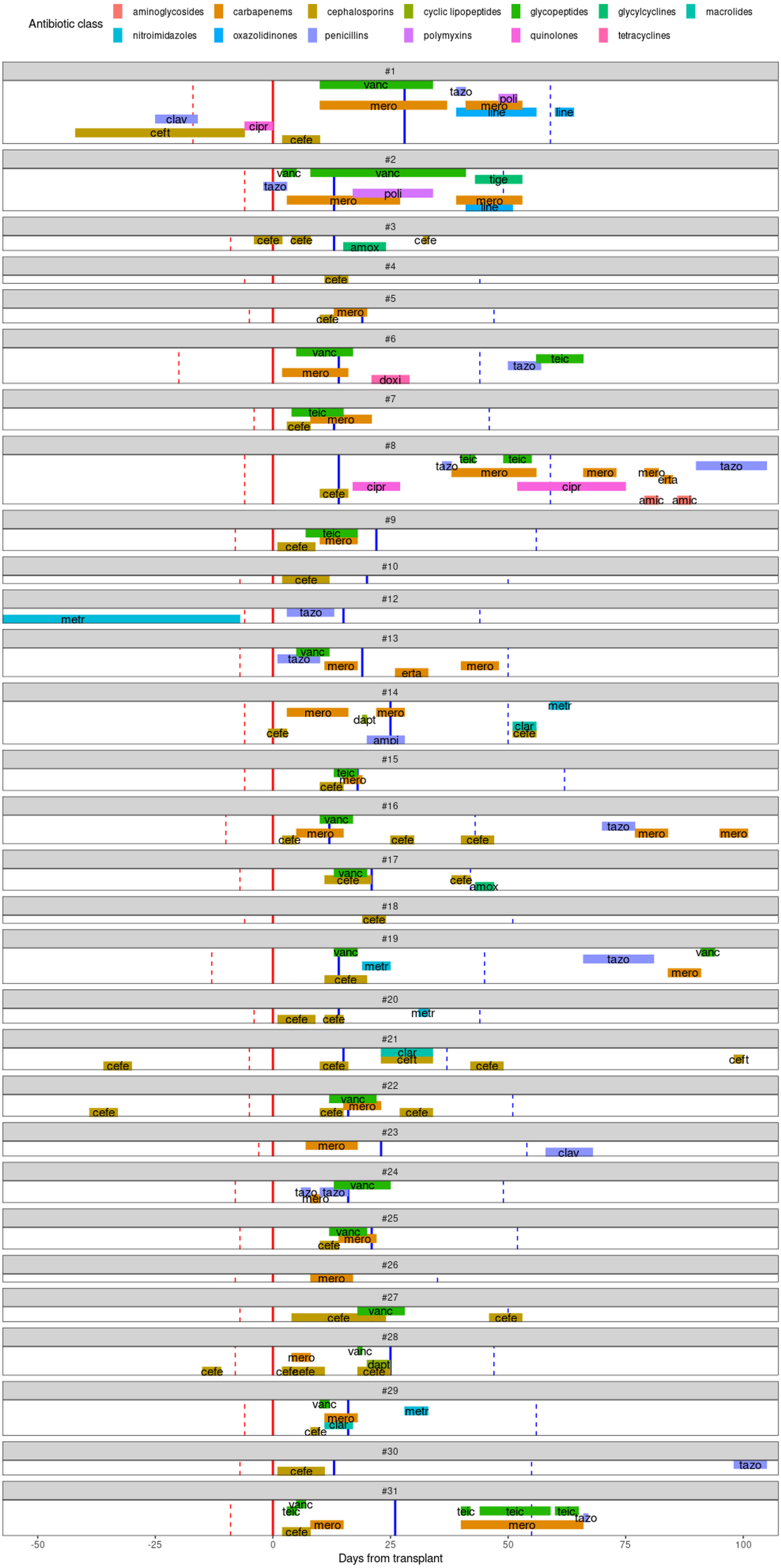

Antibiotic class
aminoglycosides
carbapenems
cephalosporins
cyclic lipopeptides
glycopeptides
glycylcyclines
macrolides
nitroimidazoles
oxazolidinones
penicillins
polymyxins
quinolones
tetracyclines
#1
vanc
tazo
poli
mero
mero
line
line
clav
cipr
ceft
cefe
#2
vanc
vanc
tige
tazo
poli
mero
mero
line
#3
cefe
cefe
cefe
amox
#4
cefe
#5
mero
cefe
#6
vanc
teic
tazo
mero
doxi
#7
teic
mero
cefe
#8
teic
teic
tazo
tazo
mero
mero
mero
erta
cipr
cipr
cefe
amic
amic
#9
teic
mero
cefe
#10
cefe
#12
tazo
metr
#13
vanc
tazo
mero
erta
mero
#14
metr
mero
mero
dapt
clar
cefe
cefe
ampi
#15
teic
mero
cefe
#16
vanc
tazo
mero
mero
mero
cefe
cefe
cefe
#17
vanc
cefe
cefe
amox
#18
cefe
#19
vanc
vanc
metr
tazo
mero
cefe
#20
metr
cefe
cefe
#21
clar
ceft
cefe
cefe
cefe
ceft
#22
vanc
mero
cefe
cefe
cefe
#23
mero
clav
#24
vanc
tazo
tazo
mero
#25
vanc
mero
cefe
#26
mero
#27
vanc
cefe
cefe
#28
vanc
mero
dapt
cefe
cefecefe
cefe
#29
vanc
metr
mero
clar
cefe
#30
cefe
tazo
#31
vanc
teic
teic
teic
teic
tazo
mero
mero
cefe
-50
-25
0
25
50
75
100
Days from transplant

Additional file 1: Timelines of antibiotic usage.

Antibiotic usage timelines for each patient in relation to stem-cell infusion. Red dashed line indicates preconditioning sampling. Red solid line indicates stem-cell infusion. Blue solid line indicates stem-cell engraftment. Blue dashed line indicates 30 days after engraftment sampling. clav, amoxicillin clavulanate; tazo, piperacillin tazobactam; amox, amoxicillin; cefe, cefepime; mero, meropenem; metr, metronidazole; ceft, ceftriaxone; vanc, vancomycin; teic, teicoplanin; cipr, ciprofloxacin; levo, levofloxacin; doxi, doxycycline; ampi, ampicillin; clar, clarithromycin; bact, sulfamethoxazole trimethoprim; erta, ertapenem; poli, polymyxin b; dapt, daptomycin; line, linezolid; tige, tigecycline; amic, amikacin.
